# Integrated Analysis of Methylome and Transcriptome Changes Reveals the Underlying Regulatory Signatures Driving Curly Wool Transformation in Chinese Zhongwei Goats

**DOI:** 10.3389/fgene.2019.01263

**Published:** 2020-01-08

**Authors:** Ping Xiao, Tao Zhong, Zhanfa Liu, Yangyang Ding, Weijun Guan, Xiaohong He, Yabin Pu, Lin Jiang, Yuehui Ma, Qianjun Zhao

**Affiliations:** ^1^ Key Laboratory of Animal (Poultry) Genetics Breeding and Reproduction, Ministry of Agriculture and Rural Affairs, Institute of Animal Sciences, Chinese Academy of Agricultural Sciences, Beijing, China; ^2^ Farm Animal Genetic Resources Exploration and Innovation Key Laboratory of Sichuan Province, College of Animal Science and Technology, Sichuan Agricultural University, Chengdu, China; ^3^ The Ningxia Hui Autonomous Region Breeding Ground of Zhongwei Goat, Department of Agriculture and Rural Areas of Ningxia Hui Autonomous Region, Wuzhong, China

**Keywords:** Zhongwei goat, deoxyribonucleic acid methylation, curly pelts, epigenetics, transcriptomics, platelet-derived growth factor C

## Abstract

The Zhongwei goat is kept primarily for its beautiful white, curly pelt that appears when the kid is approximately 1 month old; however, this representative phenotype often changes to a less curly phenotype during postnatal development in a process that may be mediated by multiple molecular signals. DNA methylation plays important roles in mammalian cellular processes and is essential for the initiation of hair follicle (HF) development. Here, we sought to investigate the effects of genome-wide DNA methylation by combining expression profiles of the underlying curly fleece dynamics. Genome-wide DNA methylation maps and transcriptomes of skin tissues collected from 45- to 108-day-old goats were used for whole-genome bisulfite sequencing (WGBS) and RNA sequencing, respectively. Between the two developmental stages, 1,250 of 3,379 differentially methylated regions (DMRs) were annotated in differentially methylated genes (DMGs), and these regions were mainly related to intercellular communication and the cytoskeleton. Integrated analysis of the methylome and transcriptome data led to the identification of 14 overlapping genes that encode crucial factors for wool fiber development through epigenetic mechanisms. Furthermore, a functional study using human hair inner root sheath cells (HHIRSCs) revealed that, one of the overlapping genes, platelet-derived growth factor C (*PDGFC*) had a significant effect on the messenger RNA expression of several key HF-related genes that promote cell migration and proliferation. Our study presents an unprecedented analysis that was used to explore the enigma of fleece morphological changes by combining methylome maps and transcriptional expression, and these data revealed stage-specific epigenetic changes that potentially affect fiber development. Furthermore, our functional study highlights a possible role for the overlapping gene *PDGFC* in HF cell growth, which may be a predictable biomarker for fur goat selection.

## Introduction

Animal hair fibers and fur are essential raw materials for the textile industry, and people have been taming and improving some fur- and wool-producing animals, such as sheep, goats, and rabbits. Unlike cashmere goats, Chinese Zhongwei goats have a reputation for their pelts, which have white, lustrous staples and attractive curls when they are obtained at approximately 35 days of age, with fibers on the skins of kids comprising 86% heterotypic fibers and 14% true wool by weight (Gong, 1994). Nevertheless, these natural and exquisite patterns are becoming less economically valuable as the curly form of the wool disappears within 2 months of the kid’s life, and the exact reasons for its disappearance remain elusive.

A few studies have found that genetic polymorphisms in candidate genes can account for various hair traits in different species (Adhikari et al., 2016; Demars et al., 2017; Morgenthaler et al., 2017). Some critical signaling pathways, including the wingless-related integration site (WNT), ectodysplasin A receptor (EDAR), and bone morphogenetic proteins (BMP) pathways, are regarded as regulatory hubs during fiber development (Lu et al., 2016; Telerman et al., 2017). Mammals, and in particular, sheep and goats have obvious periodic fiber growth with seasonal changes, and the regulatory mechanisms of these changes have been explored in transcriptome studies (Yang et al., 2017; Li et al., 2018). Subsequently, some genetic factors, such as those of the *TCHH*, *KRT* gene families, and the *metallothionein 3 isoforms*, which are related to curly wool, have been determined by RNA-seq analysis and immunohistochemical analyses of the fiber proteins (Sriwiriyanont et al., 2011; Yu et al., 2011; Kang et al., 2013). These findings may thus underpin this dynamic morphogenesis.

The epigenome, which contains a great deal of modifiable genetic information, is the source of many determining factors in regulatory mechanisms. Increased DNA methylation and histone modification status may enhance pathological immune responses and suppress hair follicle (HF) development in anagen (Zhao et al., 2012). Diverse whole genome methylation profiles have been found to characterize the two periods (anagen and telogen) of HF growth, suggesting that increased transcript expression levels are connected with compromised DNA methylation (Bock et al., 2012). Highly expressed *DNA methyltransferase 1* (*DNMT1*) can prevent the epithelial progenitor cells in the HF bulb from overproliferating to drive differentiation, thus maintaining a normal HF structure (Sen et al., 2010). Because of the tight connection between individual development (including skin development, regeneration, and HF cycling) and DNA methylation (Gudjonsson and Krueger, 2012; Botchkarev et al., 2013; Plikus et al., 2015), it is necessary to concentrate the genome-wide methylation profile of dynamic hair morphogenesis.

In the present study, we assessed DNA methylation profiles by whole genome bisulfite sequencing (WGBS) and transcriptional expression by RNA sequencing analysis (RNA-seq) of shoulder skin samples from 45-day-old kids with curly wool and from the same kids exhibiting non-curling wool at 108 days. Integrated analysis (WGBS and RNA-seq) selected differentially expressed-methylated gene (DEGs-DMGs) candidates, which tend to be the key factors in curly wool development through epigenetic patterns. Eventually, the promoting effect of the platelet-derived growth factor C (*PDGFC*) gene on HF cell growth was validated through a functional study *in vitro*. This study confirms the importance of an integrated analysis that combines DNA methylation and gene expression for determining curly hair traits and provides comprehensive resources for studying HF development in humans.

## Materials and Methods

### Animals and Sample Preparation

Three Chinese Zhongwei goats bred at the breeding base of Zhongwei goats (located in the Ningxia Hui Autonomous Region, China) were randomly selected for this study. The goats had no kinship relevant to their use as samples, and they were raised under the same conditions to minimize external factors. When they were 45 and 108 days old, we cleaned their hair and disinfected the target in the scapular region, from which skin samples were collected using sterilized scalpel blades. Some samples were immediately stored in RNAlater (Thermo Fisher Scientific, USA) for storage at −80°C until further processing, and some samples were rapidly stored in a 4% paraformaldehyde fixation solution to prepare paraffin sections. All the resulting wounds were treated with Yunnan Baiyao powder (Yunnan Baiyao Group Co., Ltd., China) to stop the bleeding. All of the animal experimental procedures were performed in accordance with the guidelines for the care and use of experimental animals established by the Ministry of Agriculture of the People’s Republic of China and approved by Institute of Animal Science, Chinese Academy of Agricultural Sciences.

### Ribonucleic Acid Isolation and Sequencing

Samples taken at 45 days and 108 days, representing curly haired (D45) and wavy haired (D108) individuals, respectively, were stored separately in RNAlater. Total RNA was extracted from these six samples by RNeasy Mini Kit (Qiagen, Germany) according to the manufacturer’s protocol. The RNA quantity and quality were assessed using an Agilent 2100 Bioanalyzer (Agilent Technologies, CA, USA), and the RNA Integrity Number value of these samples was determined to be greater than 7.5, which was important to ensure RNA integrity. The RNA library construction, quality control, and sequencing were conducted using an Illumina Nova seq platform at the Berry NGS Company (Beijing, China), through which approximately 55 million paired-end reads (2 × 150 bp) were produced for each of samples. Before the downstream analysis, filtration of sequencing reads was conducted to remove the reads containing joints and to eliminate the low-quality reads. The remaining clean data were matched to the reference genome at CHIR_1.0 (September 10, 2015, ftp://ftp.ncbi.nlm.nih.gov/genomes/all/GCA/000/317/765/GCA_000317765.1_CHIR_1.0/) using the new version of HISAT2 (v2.1.0) (Kim et al., 2015). The average alignment rate for the RNA-seq was 83.53% (82.63–84.70%, median = 83.45%) (**Supplementary Table S1**), and then, the transcripts were assembled, quantified and merged with StringTie (v1.3.4). The output files were prepared for use in the differential expression analysis. RNA-seq data were deposited into a NCBI BioProject section under accession number PRJNA524985.

### Differential Expression Analysis

Output files containing the expression levels of the exons, introns, and transcript of each sample were processed with the Ballgown R package (v2.14.1) (Frazee et al., 2015). A parametric F-test using “stattest” function in Ballgown module was used to compare transcript abundance, and the covariate “curly (samples in 45 days)/wave (samples in 108 days)” was corrected for the calculated p value. The differential expressed transcripts (DETs) were annotated to gene names to identify and list the DEGs, which were screened based on the following criteria: p value ≤ 0.05 and absolute fold change value > 1.5. The heatmap of DEG hierarchical clustering was performed using the R package pheatmap (v1.0.10).

### Differentially Expressed Genes Profile Analysis

We selected eight DEGs randomly and validated their expressions by using quantitative (qPCR) on an ABI 7500 (Applied Biosystems, USA) in a 20-μl reaction containing 2 μl of the complementary DNA template (generated by the reverse transcription kit, Takara), 10 μl of 2 × SYBR Green Master Mix (RR420A, Takara), and 0.8 μl of each primer (10 μmol/μl), with glyceraldehyde 3-phosphate dehydrogenase as the endogenous control. The primers used for qPCR were designed with Primer Premier 5 (v5.00, http://www.premierbiosoft.com) and are listed in **Supplementary Table S2**. The transcription factor binding site analysis was performed by uploading our DEG list into the Innate DB database (v5.3) (Breuer et al., 2013), where the data were subjected to the hypergeometric algorithm and with Benjamini-Hochberg correlation method (p value ≤ 0.05). A transcript splicing analysis of the DEGs was carried out by combining the extracted splice-sites information from the genome annotation results and our RNA-seq data, and we compared the alternative splicing events of each gene in two periods. Differential alternative splicing events, including skipped exon (SE), alternative 5’ splice site (A5SS), alternative 3’ splice site (A3SS), mutually exclusive exon (MXE), and retained intron (RI) events were detected using rMATS (v 4.0.1) (Shen et al., 2014), and the events were considered as significantly different based on the following filtering criteria: | Inc level difference | ≥ 5% and false discovery rate (FDR) < 0.01. To explore the potential relationships among expressed DEGs, a weighted correlation network analysis (WGCNA) was performed using the WGCNA R package (v 1.68) (Langfelder and Horvath, 2008) with 326 DEGs used as input data. We retained the genes ranked in the top 90% of the variance size between two groups, and retained 242 DEGs to generate correlation networks. The soft threshold value was set as 8. The correlation between eigenvectors of each module and the curl-wavy status was calculated, and those with |coefficient value| > 0.8 and p value < 0.05 were considered to significant.

### Whole Genome Bisulfite Sequencing

Genomic DNA (from three samples taken at 45 days, three samples taken at 108 days) were isolated from scapular skin tissues using Wizard Genomic DNA purification kit (Promega, Madison, USA) following the manufacturer’s instructions. Constructed DNA libraries were sequenced at the Berry NGS Company (Beijing, China) using an Illumina Nova seq 6000 platform (Illumina, San Diego, CA, USA), and the subsequent raw reads were filtered to remove contaminated reads in three steps: 1) removing any read that contained a 3’ adapter oligonucleotide sequence, 2) removing any read for which the percentage of Ns (unknown bases) was > 10%, and 3) removing any low quality reads (Phred score ≤ 5, percentage of low quality bases ≥ 50%). Then, an average of 600,000,000 paired-end 150-bp reads was acquired for the six samples. Next, lambda sequences were included in the clean reads to evaluate the C-T conversion rate. The sample information for methylation sequencing data were submitted to the NCBI BioProject section under accession number PRJNA555706.

### Deoxyribonucleic Acid Methylation Data Analysis

The quality controlled clean reads were converted into bisulfite-treated status reads (C-to-T and G-to-A transformed) before being aligned with the corresponding bisulfite-converted goat reference genome, CHIR_1.0 (Dong et al., 2013), using Bismark (v 0.7.12) (Krueger and Andrews, 2011). After the reads were processed as BAM files by using SAMtools (v 1.9) (Li et al., 2009), Picard software (v 1.96) was used to remove duplicate reads, and the data on methylation status per cytosine site were extracted based on the Bismark instruction manual. Methylation can be identified by determining the methylation level of a specific cytosine site according to the following formula: averaged methylation level = counts of methylated reads/counts of total reads × 100%. We then used the scriptlet “bismark_methylation_extractor” in Bismark to analyze the methylation status throughout the whole genomic DNA and the methylation distribution in various genomic regions (including upstream, downstream, gene, exonic, intronic, and intergenic regions). We identified the differentially methylated regions (DMRs, with a 500 bp window) based on the sliding window approach combined with a logistic regression method using methylKit software (v 1.10.0) to compare the methylation status of specific regions in the two groups (Akalin et al., 2012). The following screening criteria were used for obtaining the DMRs: 1) the average methylation difference between two pairwise groups was > 0.25; 2) the FDR value of methylation difference was <0.05; and 3) all DMRs were in uniquely mapped regions. The obtained DMRs were annotated to different genomic regions, where they could be considered DMGs because they had overlapping methylated cytosine (mC) sites in functional gene regions.

### Polymerase Chain Reaction Validation of the Bisulfite Sequencing

A total of 500 ng of genomic DNA previously extracted from each sample at the same time points (three samples) were mixed together and treated with bisulfite with an EZ DNA Methylation-Gold Kit (Zymo Research). The information on the primers used for bisulfite sequencing PCR is listed in **Supplementary Table S3**. Bisulfite-treated products were amplified by High Fidelity Taq DNA polymerase (Thermo Fisher Scientific) according to the manufacturer’s instructions. The PCR products purified by DNA Gel Extraction Kit (Qiagen) were ligated to a T-vector plasmid (TransGen Biotech), and the plasmids were transformed into *Escherichia* coli DH5α competent cells (Takara). We selected 10 single amplified clones for each group after they had incubated on a solid medium. Sequencing detection for the single clones was conducted by Sangon Biotech (Shanghai). Sequence data were analyzed by the online DNA methylation analysis platform at the BISMA website (http://services.ibc.uni-stuttgart.de/BDPC/BISMA/).

### Gene Functional Enrichment Analysis

We used the g:Profiler web server (v 0.6.7) (Reimand et al., 2016) to conduct the Gene Ontology (GO) enrichment analysis of the DEGs and DMGs. All genes with average expression FPKM (fragments per kilobase of transcript per million fragments mapped) > 1 from all the samples were used as the background gene set for this analysis, and GO terms in which the *P* value ≤ 0.05, as corrected by the g:SCS threshold method (a significance criterion in g:Profiler), were considered significant. An analysis of Kyoto Encyclopedia of Genes and Genomes (KEGG) pathway enrichment for the DEGs and DMGs was conducted through the online software KOBAS (v3.0) (Wu et al., 2006) to detect the related signaling pathway of each candidate gene set (BH-corrected *P* value < 0.05).

### Protein Interaction Network of Integrated Genes

We used the STRING database (v10.5) (Szklarczyk et al., 2017) to construct and screen for a protein–protein interaction (PPI) network that contained differentially methylated and expressed genes (D45 versus D108). We only retained edges of the network that meet the following parameters: confidence score >0.8 and combined score > 0.8. Cytoscape (v3.6.0) (http://www.cytoscape.org/) was used to visualize interactions for the gene-gene pair input, including their combined score and the expression and methylation trends.

### Cell Culture and Downstream Validation

Since the functional effects on fiber shape are changed, we used human hair inner root sheath cells (HHIRSCs) for further validation. We obtained HHIRSCs from ScienCell research laboratories (Carlsbad, CA, USA). The cells were incubated in mesenchymal cell medium (ScienCell Research Laboratories), which contained 1% mesenchymal stem cell growth supplement (MSCGS) (ScienCell Research Laboratories), 5% fetal bovine serum (FBS) (ScienCell Research Laboratories), 100 U/ml penicillin (ScienCell Research Laboratories), and 100 μg/ml streptomycin (ScienCell Research Laboratories) in a humidified, 37°C, 5% CO2 atmosphere, and the culture vessels were prepared with poly-L-lysine (2 μg/cm2) 1 day seeding to promote cell adherence. The cells were passaged through 4, but less than 5, population doublings to ensure their mesenchymal cell morphology and for use in other transfection experiments. After reviewing previous studies, we selected SMAD3 and *PDGFC* as candidate genes because they are potentially epigenetically regulated and act on HFs or on epidermal cell development during initial follicle formation. We conducted our pre-experiment using mouse fibroblasts (NIH/3T3 cells) to determine whether the overexpression of the selected genes had effects on the key signatures involved in the development of HFs (data not shown). We then chose *PDGFC* as the candidate to use in our further validation experiments with the human inner root sheath cells (HHIRSCs) since only *PDGFC* had a significant effect on the signatures of HFs development. The full-length coding DNA Sequence (CDS) of Homo sapiens *PDGFC* was ligated to pIRES2-EGFP to construct the overexpression plasmid, while the negative control (pIRES2-EGFP-NC) was constructed without target genes. To insert the targeted gene into HHIRSC cells effectively, we used a Lipofectamine® 3000 Transfection Kit (Invitrogen, Carlsbad, CA) following the manufacturer’s instructions. Three small interfering RNA (siRNA) sequences (RiboBio, Guangzhou, China) were used to knock down the expression of *PDGFC* in the HHIRSCs: si-h-*PDGFC*_001, CCAACCTGAGTAGTAAATT; si-h-*PDGFC*_002, GGAACAGA ACGGAGTACAA; and si-h-*PDGFC*_003, GGAAGACCTTATTCGATAT. The siRNA transfection was conducted using a specific siRNA transfection kit and riboFECT™ CP reagent (RiboBio, Guangzhou, China).

All the processed cells were collected from six-well plates after 48 h of incubation for the RNA extraction using the TRIzol method. The qPCR of the related genes was performed according to methods described above, and the primer information is listed in **Supplementary Table S2**.

For evaluating the cell motility after we conducted different treatments, we performed a monolayer wound healing assay when cells were approaching 100% confluence, a wound was made by scratching the monolayer with a pipette tip, and then, the cells were incubated in the same condition as described. After 12 h, we compared the gaps with respect to the wound line among the different groups using program ImageJ software (v 1.52a) (NIH, Bethesda, MD, USA) and calculated the migration rate by the following equation: migration rate% = [1 − (wound gap at 12 h/wound gap at T0)] × 100%, where T0 represents the initial evaluation time, which was recorded immediately after the scratch was made.

Cell proliferation at four time points after the respective cell treatment (12, 18, 24, and 36 h) was evaluated using a Cell Counting Kit (CCK)-8 assay. HHIRSCs were seeded into 96-well plates at the same density (5×103 cells per well) before transfection, and each condition was replicated in four wells independently. After adding 10 μl of CCK-8 solution (Dojindo, Kumamoto, Japan) to each well for a 2 h incubation at 37°C, the absorbance values at 450 nm were measured using a multifunctional spectrophotometer (Tecan Infinite 200 PRO, Tecan Group LTD, Austria).

## Results

**Figure 1:**
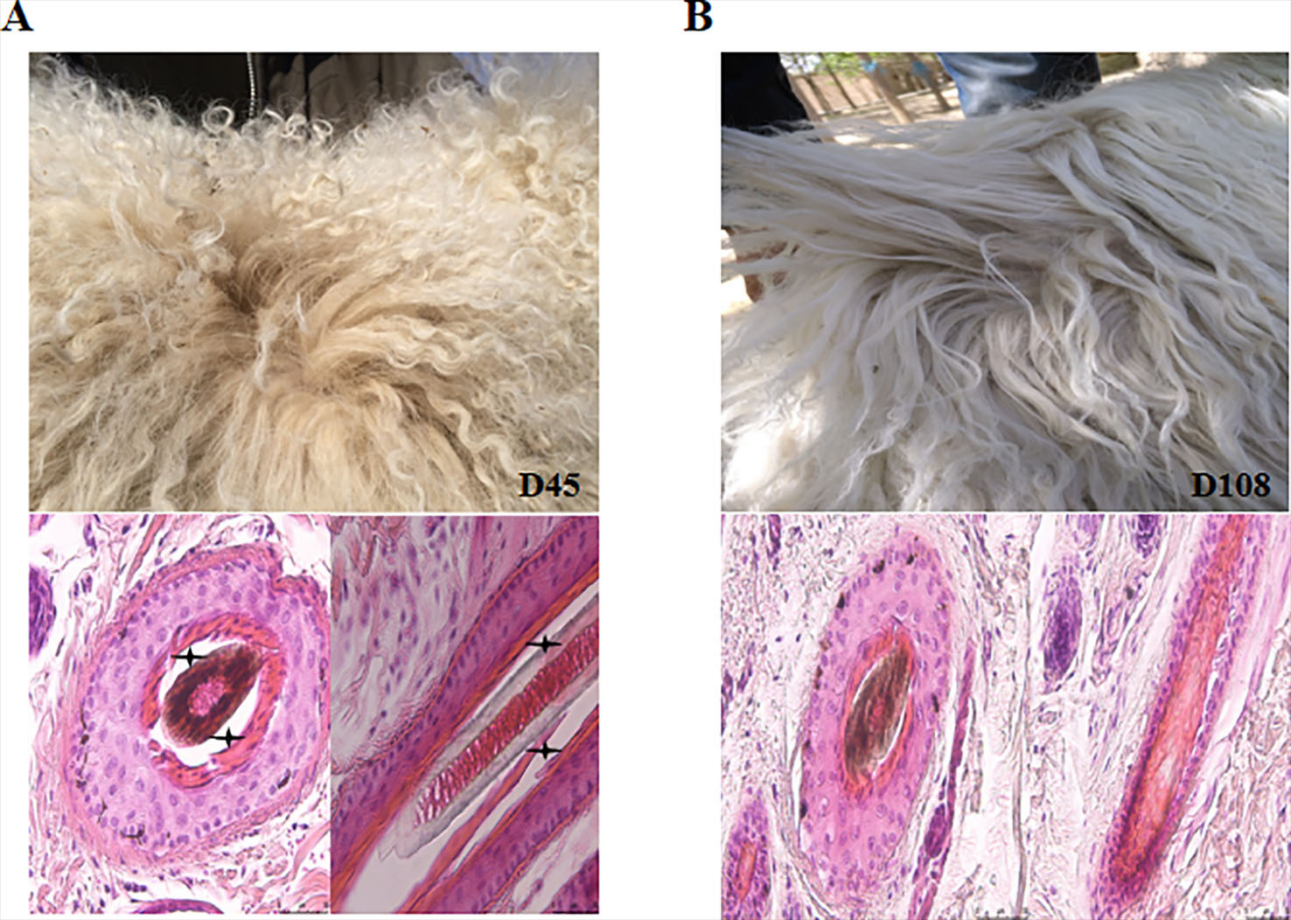
The dynamics of fleece shape. **(A)** The fur of the Zhongwei goat at 45 days old and transverse/longitudinal sections of single hair follicle. **(B)** The fur of Zhongwei goat at 108 days old and transverse/longitudinal sections of a single hair follicle. Asterisk symbol highlights the gap between the hair shaft and inner root sheath at 45 days; the bar in all images of HE stained sections is 50 μm.

To investigate the underlying mechanisms of wool fiber development in the two postnatal stages (45 and 108 days), representing curly and wavy fleece, respectively, we sampled scapular skin tissues from three unrelated Chinese Zhongwei goats at these two time point (**Figure 1**). Furthermore, we detected the HF structure at these two time points, and we found that the HF taken at 108 days had a more compact arrangement than that examined at 45 days (**Figures 1A**, **B**). We isolated DNA and RNA for further analysis by RNA-Seq and WGBS. After quality control and data refining, we obtained 22,034 gene transcripts and approximately 300 million methylation sites, which were included in our subsequent downstream analysis.

### The Messenger Ribonucleic Acid Transcriptome Reveals Distinct Signatures in Dynamic Skin Development

**Figure 2:**
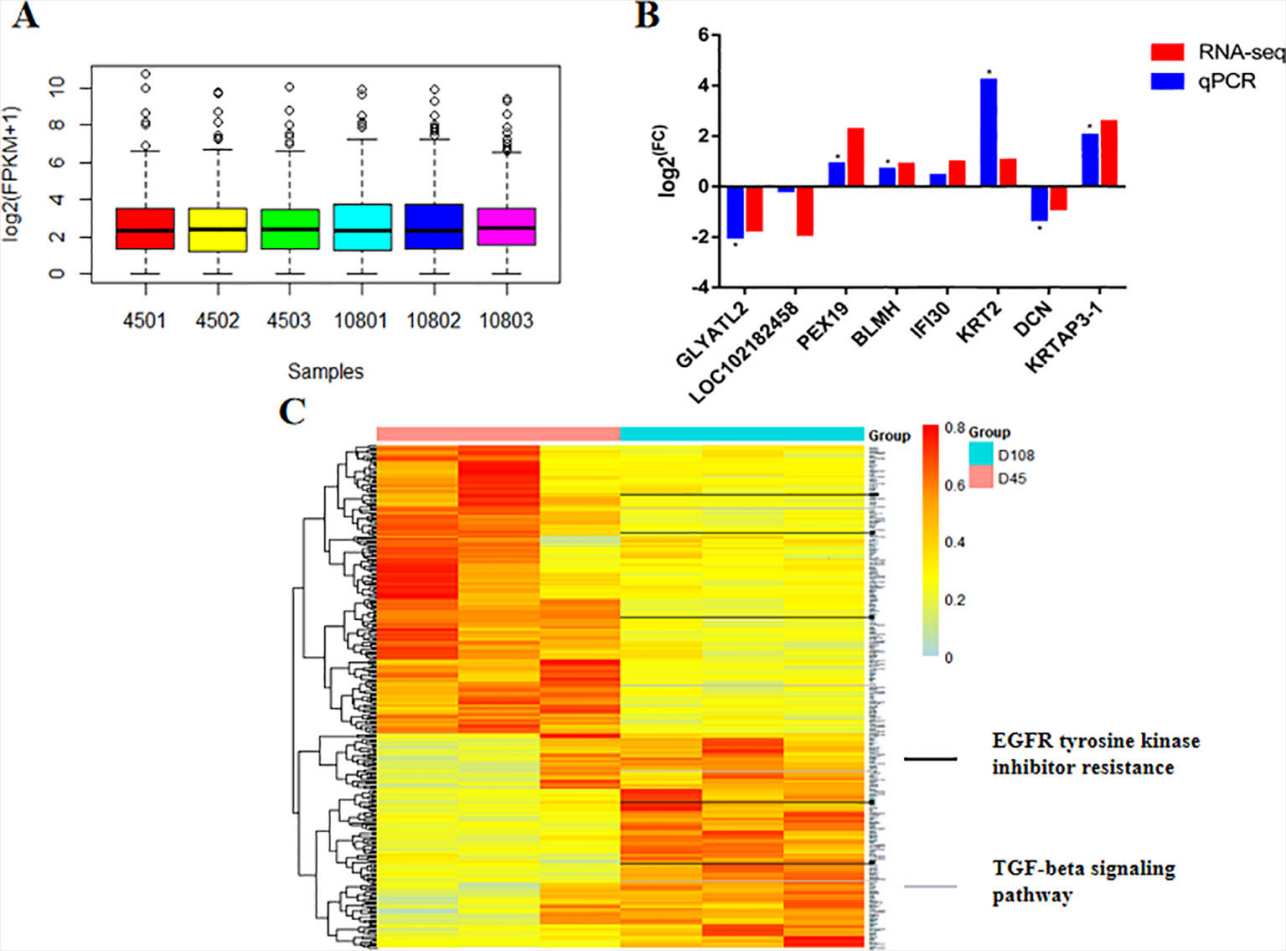
The transcriptional profile of two groups during early hair growth. **(A)** Averaged RNA expression level among six individuals. **(B)** Validation of selected differently expressed genes (DEGs) through real-time quantitative PCR. The value is presented as the logarithmic form of the fold change (FC) between two groups. *P* values were calculated using Student’s t tests (*P < 0.05). **(C)** Clustered heatmap representation of DEG expression. Clustering was performed according to Pearson’s correlation values. The black and gray bars outlined in the picture represent the DEGs that are involved in epidermal growth factor receptor tyrosine kinase inhibitor resistance and transforming growth factor beta signaling pathways, respectively.

The transcriptomic profiles obtained using RNA-seq were based on 41–42 and 38–50 million clean read pairs from the D45 and D108 samples, respectively and were uniquely mapped onto the *Capra hircus* CHIR_1.0 genome. All samples had at least 90.77% reads equal to or exceeding Q30 (**Supplementary Table S1**). These transcripts of the six samples showed a similar expression trend, with an average log2 (FPKM+1) value of approximately 2, suggesting the reliability of the general expression profiles (**Figure 2A**). For all the expression profiles, we found 326 DEGs, including 186 upregulated genes and 140 downregulated genes. We then verified the differential expression levels obtained from RNA-seq using qPCR, and the eight DEGs that were selected randomly followed the same trend as that of the sequencing data (**Figure 2B**). A hierarchical clustered map revealed that the expression of these 326 DEGs coincided with the same development period (**Figure 2C**).

**Figure 3:**
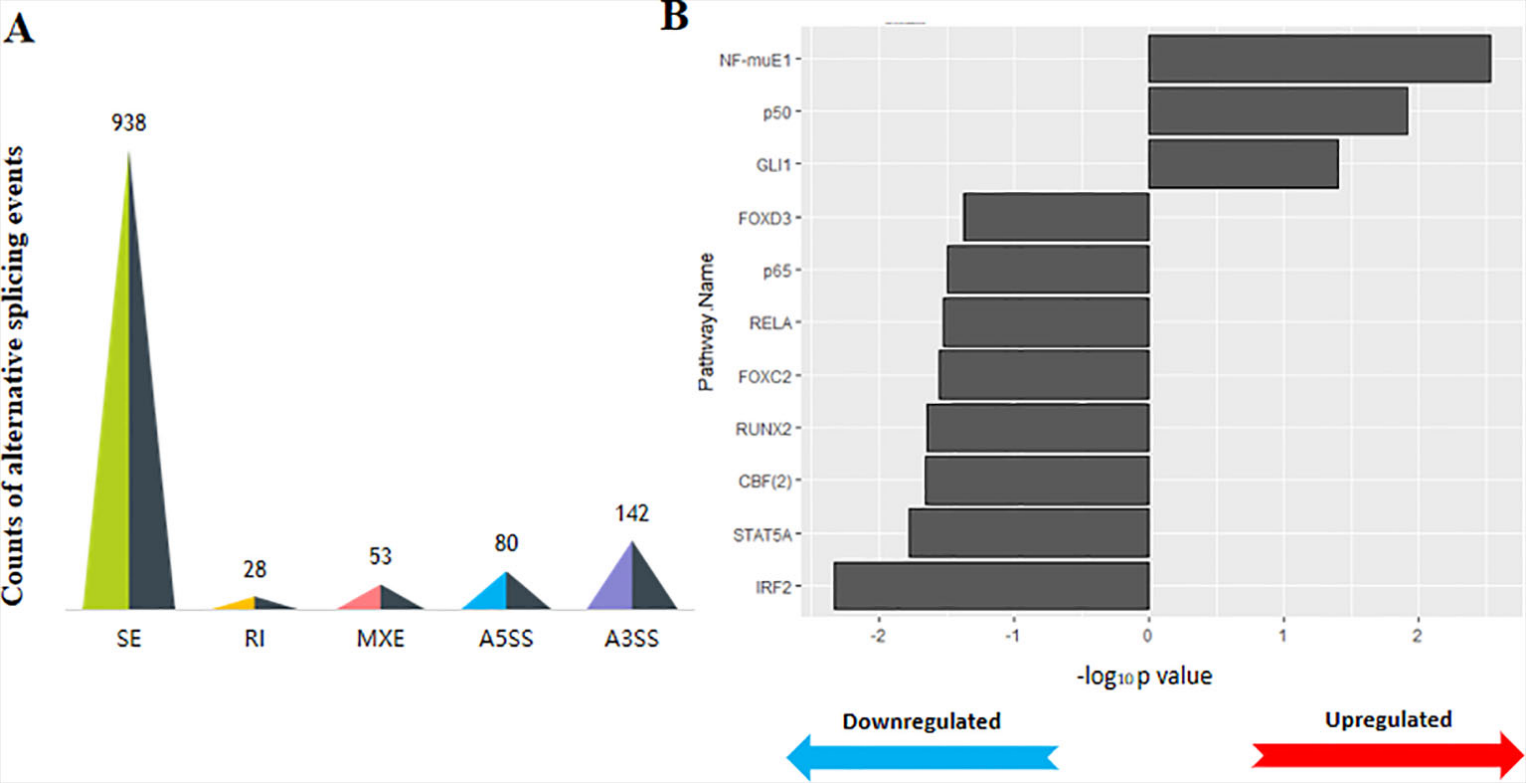
The transcriptional process is changed during wool fiber development. **(A)** Count of the differential splicing events between D45 and D108 transcripts (*P* < 0.05). SE, skipped exon; RI, retained intron; MXE, mutually exclusive exons; A5SS, alternative 5’ splice site; A3SS, alternative 3’ splice site. There are 938 significantly different skipped exon events between the two stages, which was much greater than other events. **(B)** Transcription factor binding sites enriched among differentially expressed genes in D45 and D108. Only the transcription factors of upregulated or downregulated genes that had a *P* value < 0.01 were retained. All *P* values were corrected using the Benjamini–Hochberg method (BH-corrected *P* value < 0.05).

We further explored whether the developmental process of HF affected alternative splicing events. We determined the distribution of the splicing events among the two groups and found no significant differences (**Supplementary Figure S1**), while the DEGs had a higher number of SE events (938, FDR < 0.01, Inc level difference > 5%) that were significantly different between the two groups (**Figure 3A**). A transcription factor (TF) enrichment analysis was performed to investigate the regulatory networks among these DEGs connected by coreactive TFs. For example, transcription factor *GLI family zinc finger 1 (GLI1)* and *interferon regulatory factor 2 (IRF2)* were significantly enriched in the promoters of upregulated and downregulated genes, respectively (**Figure 3B**).

To better understand the functions of these DEGs, a GO enrichment analysis was performed. The top five GO terms were cytoplasmic part (GO: 0044444), cytoplasm (GO: 0005737), cytosol (GO: 0005829), skin development (GO: 0043588), and cornification (GO: 0070268) (**Supplementary Table S4**), which highlight the central roles of the cell conformation and keratinization in the DEGs during hair structure transformation. In the KEGG pathway enrichment, the antigen processing and presentation (chx04612), systemic lupus erythematosus (chx05322), epidermal growth factor receptor (EGFR) tyrosine kinase inhibitor resistance (chx01521), fatty acid elongation (chx00062), asthma (chx05310), inflammatory bowel disease (chx05321), leishmaniasis (chx05140), selenocompound metabolism (chx00450), transforming growth factor beta (TGF)-beta signaling pathway (chx04350), and FoxO signaling pathway (chx04068) were considered significantly enriched pathways (**Table 1**).

**Table 1:** Kyoto Encyclopedia of Genes and Genomes pathway enrichment of differentially methylated genes and differently expressed genes.

KEGG pathway of DMGs (IDs)	Number of DMGs involved	P value	Overlapped genes	KEGG pathway of DEGs (IDs)	Number of DEGs involved	P value
Glutamatergic synapse (chx04724)	21	8.98054E-07		Antigen processing and presentation (chx04612)	6	0.001234391
Adherens junction (chx04520)	15	2.05829E-05		Systemic lupus erythematosus (chx05322)	7	0.004038687
Inflammatory mediator regulation of TRP channel (chx04750)	17	5.60775E-05		EGFR tyrosine kinase inhibitor resistance (chx01521)	5	0.007151845
Axon guidance (chx04360)	23	8.68786E-05		Fatty acid elongation (chx00062)	3	0.008275261
Long-term depression (chx04730)	12	9.18081E-05	SMAD3,*PDGFC*	Asthma (chx05310)	3	0.01251151
Oxytocin signaling pathway (chx04921)	21	9.7458E-05		Inflammatory bowel disease (chx05321)	4	0.016331757
Arrhythmogenic right ventricular cardiomyopathy (chx05412)	13	0.000158767		Leishmaniasis (chx05140)	4	0.023108939
Aldosterone synthesis and secretion (chx04925)	14	0.000169753		Selenocompound metabolism (chx00450)	2	0.026689839
Rap1 signaling pathway (chx04015)	23	0.000689252		TGF-beta signaling pathway (chx04350)	4	0.029163353
Gap junction (chx04540)	12	0.002506112		FoxO signaling pathway (chx04068)	5	0.037441899

Overlapped genes, the genes that are enriched in significant pathways as both DMGs and DEGs.

**Table 2:** Summary of the whole-genome bisulfite sequencing dataset.

Sample	Clean reads	Unique mapped reads	Unique mapped ratio (%)	Average depth	Conversion rate (%)	%≥Q30
D4501	603,524,580	442,523,036	73.32	19.86	98.84	91.97; 88.77
D4502	598,727,366	442,986,932	73.99	18.93	98.78	91.96; 87.98
D4503	601,082,668	445,831,838	74.17	20.62	98.87	91.96; 88.79
D10801	505,312,936	360,303,746	71.30	16.63	98.73	92.01; 88.50
D10802	604,767,486	440,587,260	72.85	20.00	98.86	91.83; 89.14
D10803	614,904,624	459,049,754	74.65	23.03	98.91	91.77; 88.92

Based on the WGCNA, we derived associated DEG expression coherence sets. There were 242 DEGs remaining after the expression comparison, blue (|coefficient of correlation| = 1, *p*-value = 2×10^−5^) and turquoise (|coefficient of correlation| = 0.82, *p*-value = 5×10^−2^) modules were correlated with curly wave status. The results indicated 91 and 151 genes were in the blue and turquoise modules, respectively (**Supplementary Figure S2**). We considered that a weight of gene-gene edges greater than 0.4 indicated a stable correlation, which led to the blue module having 255 edges and the turquoise module having 174 edges. A KEGG pathway enrichment analysis was also performed to determine the gain-the-function assessment of the DEGs in the two modules. Both modules were significantly enriched in EGFR tyrosine kinase inhibitor resistance and the PI3K-Akt signaling pathway (data not shown).

### The Deoxyribonucleic Acid Methylation Profile Potentially Affects the Dynamic Transformation of Wool Fibers

Bisulfite sequencing enabled the acquisition of the genome-wide DNA methylation landscapes at single-base resolution of the postnatal D45 and D108 skin samples from Zhongwei goats. We obtained 360–459 million uniquely mapped reads among all the samples to ensure concordant coverage. The average ratio of uniquely mapped reads was 73.38% (71.30–74.65%, median = 73.66%), and the sequencing depths were all greater than 16 (**Table 2**).

**Figure 4:**
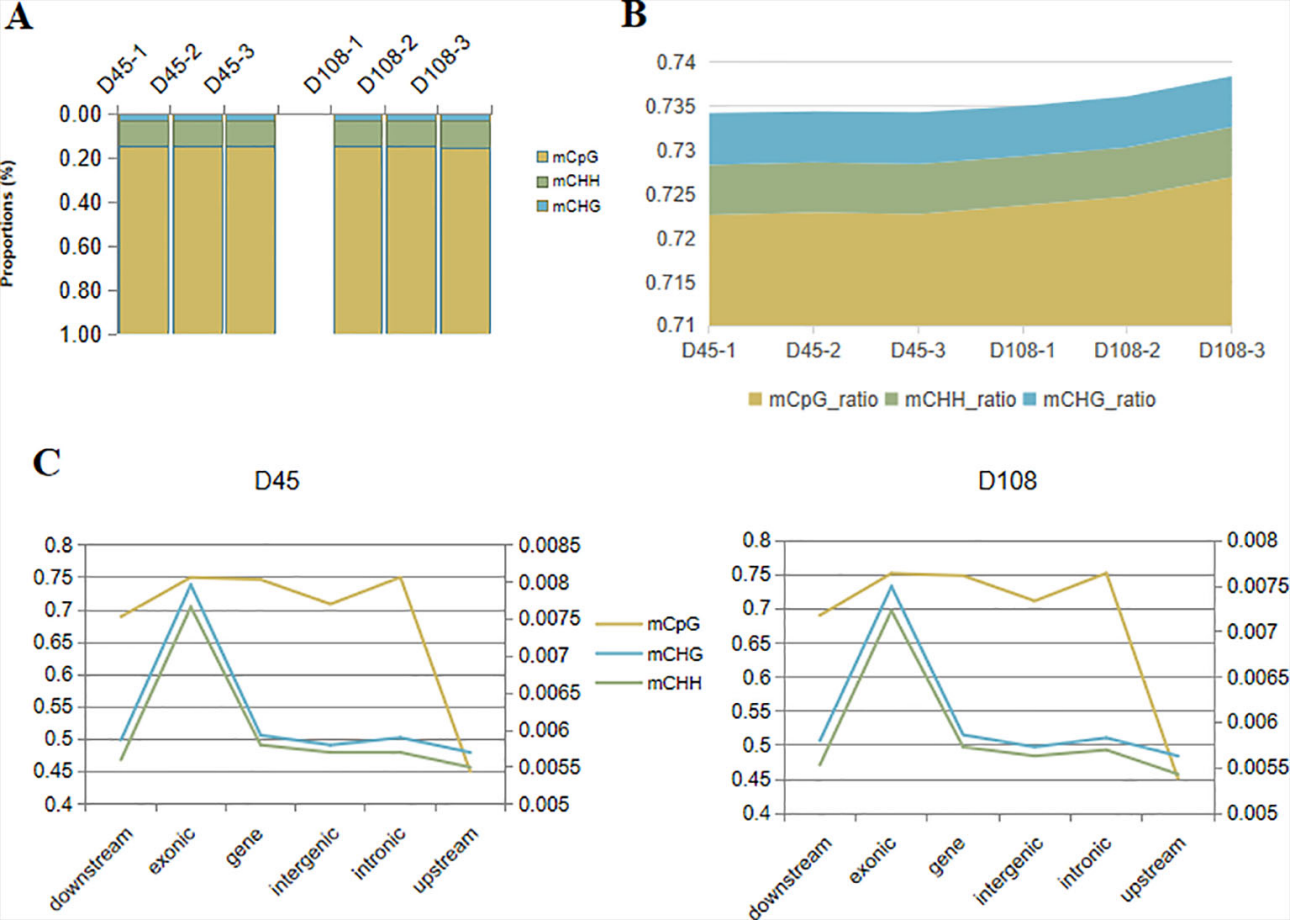
DNA methylation profiles of skin samples in shift stages in the wool shape of Zhongwei goats. **(A)** The proportion of methylated cytosines (mCs) (mCpG, mCHH, and mCHG) in the D45 and D108 tissue samples. **(B)** The average methylation level (%) of cytosine sites [CpG, CHH, and chlorhexidine gluconate (CHG)] in six individual samples. **(C)** The methylation level (%) of three mCs in different genomic regions or elements. The vertical axis on the left represents the methylation levels of CpG in two stages, and the values on the right axis represents methylation levels of CHG and CHH.

The methylation level was calculated with the average of 321,036,397 and 322,677,466 methylated cytosines (mCs) in the 45- and 108-day stages, respectively. Among these detected cytosine sites, CpG (CG sites), as one in a nucleotide context, made up the highest proportion (84.71–85.34%), while chlorhexidine gluconate (CHG) accounted for the smallest (3.47–3.58%) (**Figure 4A**). In addition to the broadest methylation distribution, these CpG sites had the highest average methylation level (72.26–72.69%), compared with the dramatically low methylation statuses found for the CHG (where H can be A, T or C) and CHH sites (0.56–0.59%) (**Figure 4B**). Although there was no obvious difference in methylation level between the two stages, we observed that, in distinct genomic features (downstream and upstream of the genes, exons, genes, intergenic regions, and introns), the regions upstream of the genes were weakly methylated at both CpG (< 50%) and non-CpG (< 0.6%) sites in all the samples, while exonic regions were the most methylated sites (CpG > 75% and non-CpG> 0.7%) (**Figure 4C**).

**Figure 5:**
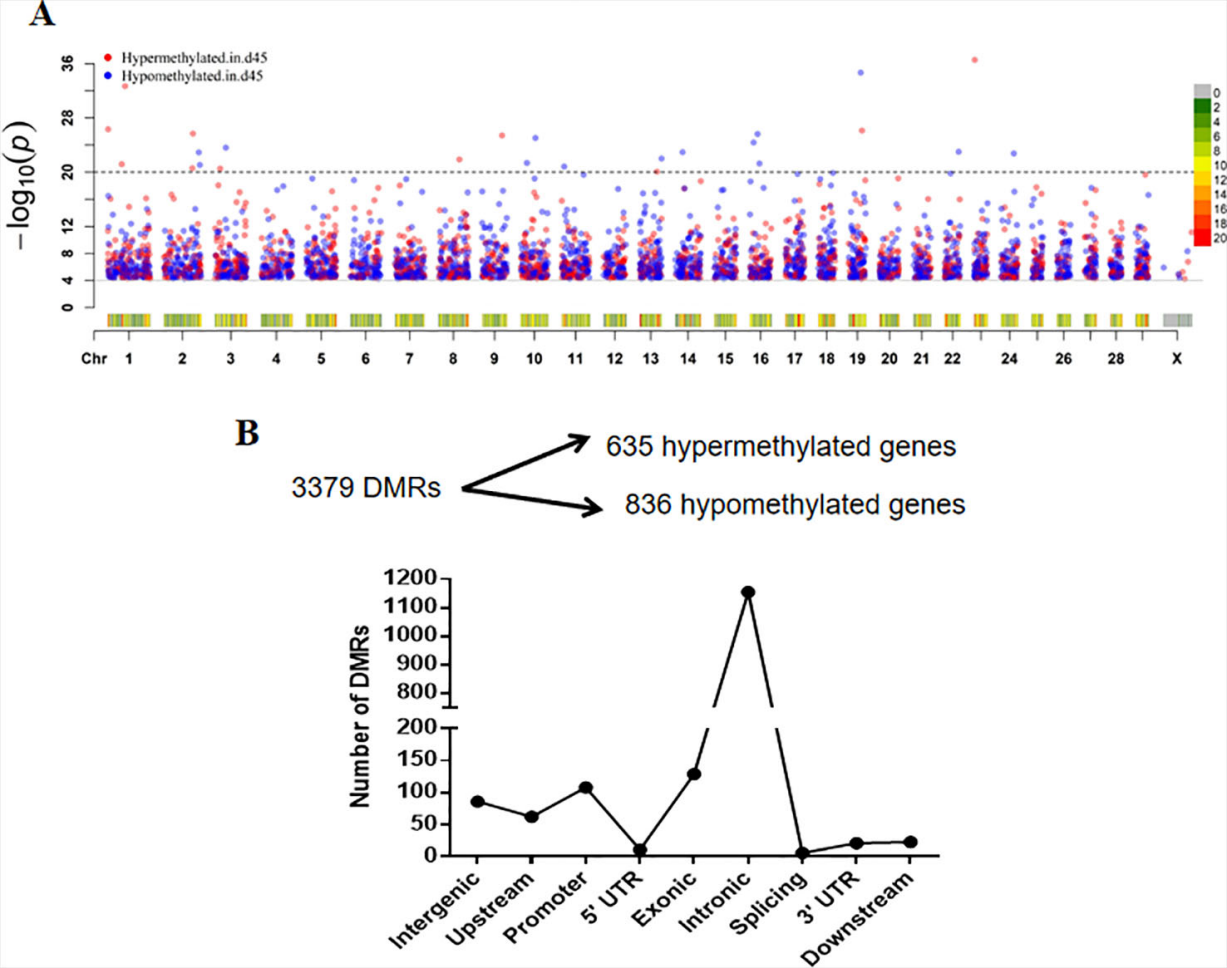
Distribution of differentially methylated regions (DMRs) in different categories. **(A)** Manhattan plot of DMRs in a chromosomal landscape. Dots above the dotted line presented DMRs with –log10 (*p*) > 20. The heatmap below the dots represents the density (counts) of the DMR distribution within chromosomes. The blue and red dots indicate the status of hypomethylation and hypermethylation in the D45 regions, respectively. **(B)** The filtration of differentially methylated genes and the number of DMRs in different genomic regions.

To identify the DMRs, we first computed the methylation status by analyzing the 500 bp-long sliding windows using the output of a methylKit (Akalin et al., 2012). A total of 3,379 DMRs were identified, including 1,651 hypermethylated DMRs and 2,128 hypomethylated DMRs in the 45-day sample (**Supplement Table S5**). A Manhattan plot was generated to show the DMR distribution along 30 chromosomes as −log10 (P-values) for all sliding windows (**Figure 5A**). The number of DMRs was reduced along with the number of chromosomes, but the DMR distribution was not affected, and a high density of DMRs was detected on chromosomes 13, 17, and 19. There were 1,471 DMRs annotated by gene name based on CHIR 1.0 assembly identification (Dong et al., 2013). We obtained 1,250 DMGs after merging data of the DMRs in the same gene, which contained 108 DMRs that were located in gene promoter regions [we considered areas upstream of the transcriptional start site (TSS) within 2,000 bp and downstream of the TSS within 200 bp as promoter regions]. Among all these DMGs, 635 were hypermethylated DMRs and 836 were hypomethylated DMRs, and 1,155 DMGs were annotated within intronic regions (**Figure 5B**). To examine the stability of the obtained DMRs, we randomly selected four DMRs that had region annotations (two intergenic, one exonic, and one upstream) of gene positions to validate the DNA methylation level. Bisulfite sequencing PCR (BSP) was used to detect the DNA methylated sites, although there was no significant difference in methylation between the samples taken at different time points, they presented similar trends in terms of methylation changes compared to those identified by the WGBS data (**Supplementary Figure S3**).

To explore the potential relationship of GO terms with these DMGs, a GO enrichment analysis was conducted by dividing these DMGs into hypermethylated and hypomethylated groups (**Supplementary Figure S4**). We identified the top 10 terms in three areas (cellular component, CC; biological process, BP; molecular function, MF), with specific descriptions, such as cell junction (GO: 0030054), cytoskeletal protein binding (GO: 0008092), cell development (GO: 0048468), cytoskeletal protein binding (GO: 0008092), and channel activity (GO: 0015267). For the KEGG pathway enrichment analysis performed on KOBAS v.3.0, we regarded adherens junction (chx04520) and gap junction (chx04540) as vital discoveries that illustrated the altered intercellular communication due to changes in epigenetic modification (**Table 1**).

### An Integrated Analysis of the Differently Expressed Genes and Differentially Methylated Genes Was Used to Identify Candidate Genes That Control Hair Morphogenesis

**Figure 6:**
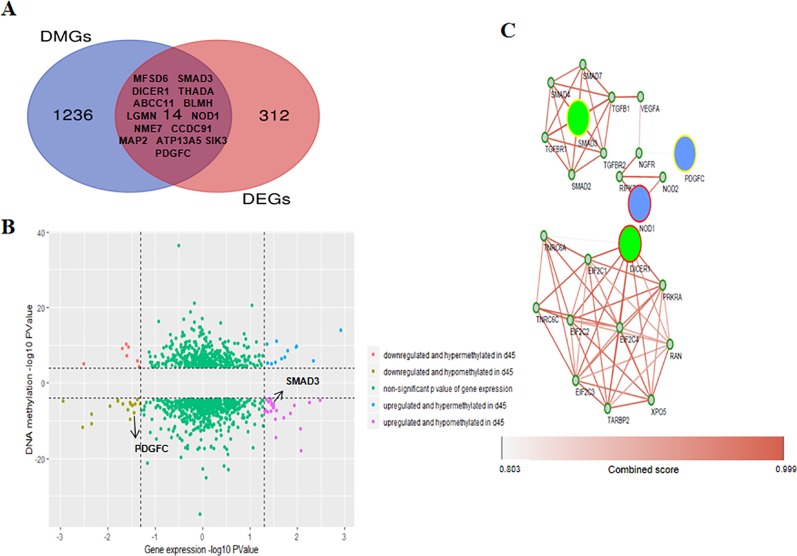
Integrated analysis was used to identify genes with coupled differential DNA methylation and RNA transcription. **(A)** Venn diagram representing methylation-modified differently expressed genes during wool transformation. **(B)** Quadrant plot showing differentially methylated genes and expression levels of the corresponding genes. The vertical dotted lines indicate a threshold of the *P* value below 0.05, and parallel dotted lines show a threshold of the *P* value below 10^−4^. **(C)** The protein-protein interaction (PPI) network among overlapping genes. Four genes identified from our analysis are included because both the confidence score and combined score were greater than 0.8. Circles filled with blue and green indicate downregulated and upregulated genes in D45, respectively. Circles with yellow and red margins indicate hypomethylation and hypermethylation in D45, respectively. Smaller gray circles with green margins denote genes attributed to the interaction.

To gain deeper insight into the RNA expression and DNA methylation differences linked to hair shaft development, we identified 14 overlapping genes among the 1,250 DMGs and 326 DEGS (**Figure 6A**). In these 14 overlapping genes, 9 annotated genes had differential methylation in the intronic regions, 4 genes were differentially methylated in intergenic areas (*SMAD3, CCDC91, MAP2,* and *SIK3*), and only 1, *LGMN,* had a DMR in the 3’ UTR. We used 1,250 DMGs and their DNA methylation and gene expression data to explore their potential correlation. We found that 31 DMGs had a negative relationship (red and purple dots in **Figure 6B**), while 29 DMGs were positively regulated (lime green and blue dots in **Figure 6B**), and the DNA methylation status of 7 overlapping genes were associated with negatively regulated expression (*THADA, NOD1, MAP2, BLMH, LGMN, SMAD3,* and *SIK3*). Moreover, we conducted KEGG pathway enrichment to uncover the signaling pathway of DEGs and DMGs, and two genes, *SMAD3* and *PDGFC*, were involved in both of the most significantly enriched pathways of the DEGs and DMGs (**Table 1**). Then, we introduced a protein interactional network into these 14 overlapping genes to explore the mutual effects on the proteomics of hair developmental processes. Overall, four genes were found to apply to the scoring criterion that we set to ensure a strong association (confidence score >0.8 and combined score > 0.8) (**Figure 6C**). Two clusters are shown in this network analysis, which has had three integrated genes *(PDGFC, SMAD3,* and *NOD1*) in one network, and *DICER1* consists of an independent cluster with other imputed associated genes. The pathway analysis of the interactive network was performed, and 14 significant pathways were found, and we noticed some canonical signaling pathways, such as the TGF-beta signaling pathway, Hippo signaling pathway and FoxO signaling pathway (**Supplementary Table S6**).

### *PDGFC* Is Associated With the Inner Root Sheath Cell Mesenchymal Phenotype and Enhances Hair Follicle Formation

**Figure 7:**
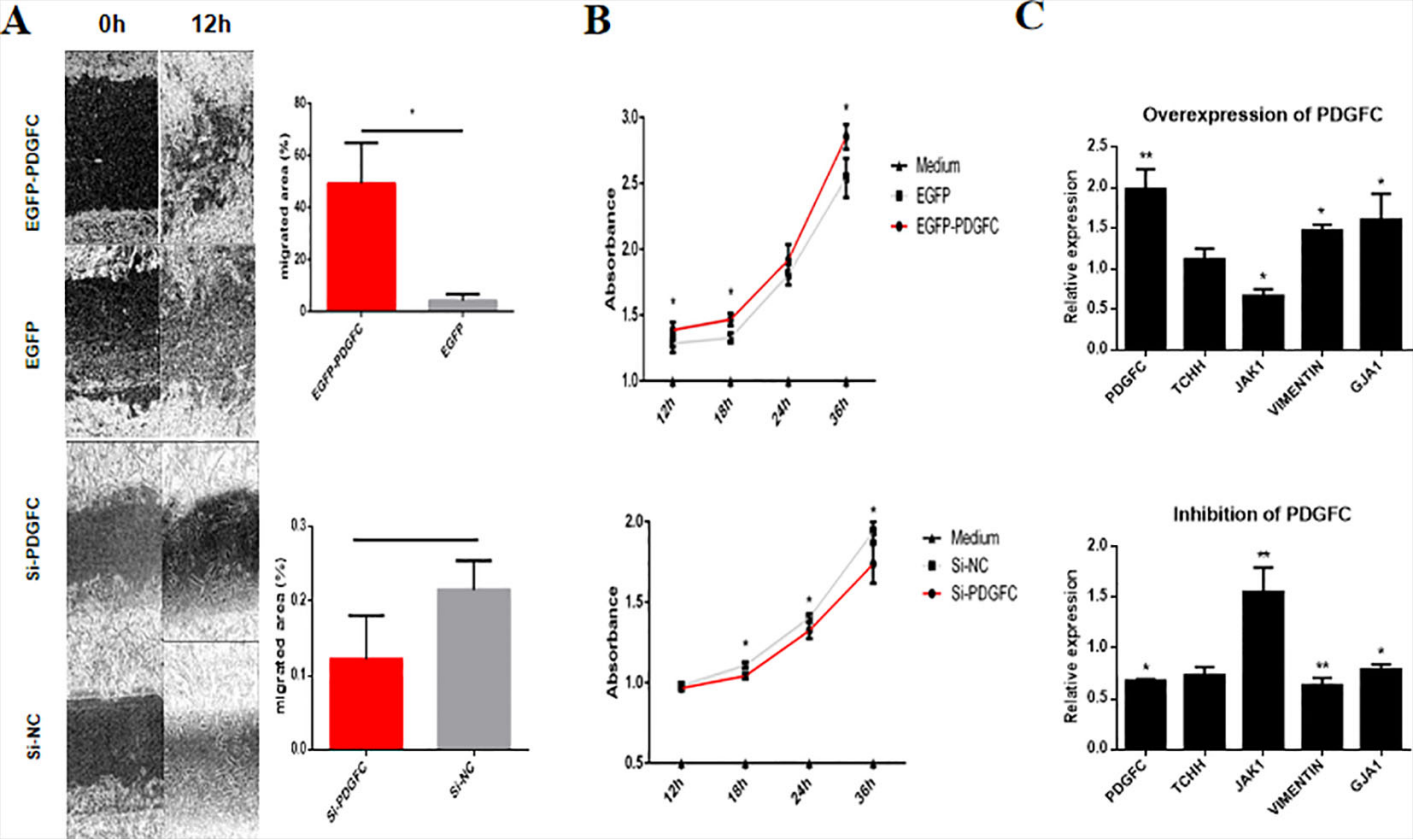
The *PDGFC* gene affects human hair inner root sheath cell (HHIRSC) migration and proliferation by regulating hair follicle-related gene expression. **(A)** A wound healing assay was conducted after the transient transfection of *PDGFC* vectors or si-*PDGFC*. Wound closure was monitored after 12 h, and the calculation of the wound closure area is explained in the methods. The mean ± standard deviation (n = 3), *P < 0.05 (two-sided t-test). **(B)** The cell proliferation rate was evaluated at four time points using the Cell Counting Kit-8 assay after the corresponding cell treatments. The mean ± standard deviation (n = 3), *P < 0.05, (two-sided t-test). **(C)** Results from the quantitation of gene expression related to hair development by quantitative PCR in HHIRSCs at 48 h after transfection with control (scrambled) or the *PDGFC* vector or si-*PDGFC*. N = 3; *P < 0.05, **P <0.01 (two-sided t-test).

To explore the role of *PDGFC* in the dynamic nature of HFs, cell culture, and overexpression, siRNA transfection was conducted using HHIRSCs. A wound healing assay showed that the HHIRSCs containing enhanced green fluorescent protein (EGFP)-*PDGFC* vectors had significantly stronger migration ability than the control group cells (EGFP) (**Figure 7A**). Correspondingly, cells with a specific RNA interference sequence of *PDGFC* exhibited relatively smaller migration areas compared with those of the group transfected with random small fragment sequences (**Figure 7A**). The cell proliferation rate was measured using CCK-8 assays, and groups with lower *PDGFC* expression (EGFP and si-*PDGFC*) showed a slower proliferation ratio at four selected time points compared with the case groups (**Figure 7B**). Furthermore, key genes associated with HF activation and development, for example, *GJA1* and *JAK1,* were upregulated and downregulated after *PDGFC* overexpressing gene transfection (**Figure 7C**). Notably, the mesenchymal marker *Vimentin* was upregulated in HHIRSC to maintain the morphologic characteristics of IRS. Not surprisingly, the suppression of *PDGFC* presented an opposite result *versus* the overexpression group (**Figure 7C**).

## Discussion

While previous studies have determined the effects of selected aspects, such as the genome-wide locus, DNA methylation, transcriptional signatures, and morphology, on the morphogenesis of curly fibers (Hynd et al., 2009; Cheng et al., 2010; Sriwiriyanont et al., 2011; Espada et al., 2014; Fan et al., 2015; Sennett et al., 2015; Gao et al., 2016; Glover et al., 2017; Li et al., 2018; Petridis et al., 2018), a comprehensive study conducting the interplay of genome-wide DNA methylation and transcription synchronously using skin tissues from the same biological replicates in different growth stages had thus far been lacking. The functional regulation of DNA methylation on gene expression has now been established as an effective prospective way to understand the ways that drastic methylation changes relate to hair phenotypic variation (Guo et al., 2014).

Overall, we observed 326 DEGs, some involved in EGFR tyrosine kinase inhibitor resistance, and the TGF-beta signaling pathway had been highlighted as valuable candidates for regulating HF development, based on previous studies (**Figure 2C**) (Cheng et al., 2010; Rognoni et al., 2014; Glover et al., 2017; Tripurani et al., 2018). Evidence from the transcription factor (TF) binding site analysis of the DEGs revealed the several TFs combine with the promoters of selected genes. For example, *interferon regulatory factor 2* (*IRF2*) and *signal transducer and activator of transcription 5A (STAT5A)* are reported TFs that serve as mediators to regulate transcriptional processes in HF growth and skin disease (Nishio et al., 2001; Legrand et al., 2016). GO terms associated with the DEGs were acquired, and the genes related to epidermal cell development were included in the five most significant terms, which intriguingly, included genes related to skin development (GO: 0043588, *TGM5, KRT23, PTCH2, S100A7, GRHL3, KRT84, MYSM1, KRT80, ACER1, KRT72, KRT2, LIPM, DSG4, SPRR4, KRTAP15-1, NF1, KRT40,* and *KRTAP3-1*) and cornification (GO: 0070268, *TGM5, KRT23, KRT84, KRT80, KRT72, KRT2, LIPM, DSG4,* and *KRT40*) (**Supplementary Table S4**), which had been closely associated with the formation of curly HFs or hair shafts in previous studies (Westgate et al., 2017). EGFR tyrosine kinase inhibitor resistance and the PI3K-Akt signaling pathway were enriched in the WGCNA modules calculated, which have associations with wavy hair coat and curly whiskers in mice (Cheng et al., 2010) and the hair cycle in many species (Kobielak et al., 2007; Feutz et al., 2008; Nie et al., 2018), respectively.

In the present study, we discovered that exonic regions showed relatively higher methylation levels compared with other regions in all nucleotide contexts (CpG, CHG, and CHH sites) (**Figure 4C**). Additionally, altered methylation profiles may induce changes to the mediation of alternative splicing through methyl-binding domain proteins (MBDs), which can regulate splicing factors indirectly (Gelfman et al., 2013). Therefore, it is reasonable to conclude that DNA methylation may regulate early HF development by mediating RNA expression and expanding the coding capacity of genes; however, this hypothesis still needs to be supported through further exploration. Among the 3,379 significant DMRs, 1,250 DMR-related genes were identified, the majority of which annotated at intronic regions (**Figure 5B**). The GO functional analysis in our study demonstrated that these DMGs were mainly enriched in the classifications of cell structure (e.g., cell projection and plasma membrane) and cell communication (e.g., cytoskeletal protein binding, channel activity and ion channel activity) (**Supplementary Figure S4**). Cell junctions (GO: 0030054) and cytoskeletal protein binding (GO: 0008092) have been reported to regulate cortex cell movement and reshaping in certain areas of the HF (Morioka et al., 2006; Harland and Plowman, 2018). These results were in agreement with previous reports and verified the importance of intercellular communication during cell reshaping of the HF bulb (Runswick et al., 2001; Arita et al., 2004).

To characterize the correlation between gene methylation and expression levels, we further focused on identifying differentially methylated (DMR-associated) and DEGs through DNA methylation profile and RNA-seq data. This integrated analysis led to the identification of 14 overlapping genes (*MFSD6, SMAD3, DICER1, THADA, ABCC11, BLMH, LGMN, NOD1, NME7, CCDC91, MAP2, ATP13A5, SIK3,* and *PDGFC*) (**Figure 6A**). Based on the KEGG pathway analysis of the DEGs and DMGs, we found that *PDGFC* and *SMAD3* were involved in the signaling pathways identified, which include roles in gap junction, EGFR tyrosine kinase inhibitor resistance, TGF-beta signaling and adherens junction, that are essential for proliferation, differentiation, and communication of HF cells during movement (Young et al., 2003; Arita et al., 2004; Plasari et al., 2010; Oshimori and Fuchs, 2012; Gay et al., 2015; Flores et al., 2018). According to the PPI network, regulated correlation between overlapping genes was detected, with 4 of 14 overlapping genes retained in the network. Among them, SMAD family members, including *SMAD2*, *SMAD3*, *SMAD4*, and *SMAD7*, have a strong relationship to protein regulation and have been reported and validated to function in the initiation of HF cycling through conventional signaling pathways (Alimperti et al., 2012; Oshimori and Fuchs, 2012; Wang et al., 2017). The overlapping gene *NOD1*, a gene implicated in the immune response that is known to obstruct bacterial invasion and initiate inflammation, as well as exert inhibitory effects on tumor cell viability and proliferation (Velloso et al., 2018).

For validation of the downstream effects, we found that higher *PDGFC* expression promoted cell migration and proliferation, a finding that is consistent with the conclusion that tight HF inner root sheath structure surrounding the HF cortical cells can promote hair growth (Langbein et al., 2003; Basmanav FB et al., 2016). The overexpression/suppression of *PDGFC* in HHIRSC also altered the RNA expression of four key factors (*TCHH*, *JAK1*, *VIMENTIN*, and *GJA1*) that are involved in related HF-regulating pathways. The mesenchymal marker *VIMENTIN* was upregulated in *PDGFC*-overexpressing HHIRSCs, a finding that supports the result from the cell proliferation assay showing increasing proliferation rates (**Figures 7B**, **C**). As a factor in EGFR tyrosine kinase inhibitor resistance signaling, *JAK1* is a molecule known to perturb skin hemostasis-related pathways and induce epidermal inflammation and downstream genes associated with clinical skin diseases (Jin et al., 2014; Jabbari et al., 2015; Yasuda et al., 2016). *GJA1* encodes the gap junction protein connexin 43 (Cx43) in nearly every tissue in the body, and high expression leads to increased connexin density, which has been observed in the proximal bulb of the IRS (Flores et al., 2018). Intriguingly, we did not find a significant expression change in the expression level of *TCHH*, which is a key gene that interacts with IRS keratins that contributes to the “hardening” process that molds hair fiber shape. We hypothesized that the low differentiation level of the HHIRSCs in our study resulted in the lack of keratin production, thus affecting the initiation of *TCHH* expression, as *TCHH* only appears where a hardened keratin structure is needed (O’Keefe et al., 1993). These results highlight the potential role of *PDGFC* in interacting with key regulators of HF development and in initiating epidermal cell proliferation to complete HF structure.

As our DNA methylomes and transcript profiles are derived only from shoulder skin showing significant trends in fur structure, other parts of the skin tissues should be further assessed by taking measurements at more time points and by excluding possible interfering factors during the developmental stage. In addition, the effect of *PDGFC* targeted to the HF formation signatures on the hair shape transition *in vivo,* together with our data, may explain these dynamic fiber changes more convincingly.

## Conclusions

This study determined the role of methylation dynamics in the curly fleece transition of the Chinese Zhongwei goat. The profile of DNA methylation and gene expression was affected among the kids during postnatal development, suggesting that epigenetic processes contribute to the developmental transitions largely driven by regulating related biological factors. We identified 1,250 DMGs that mainly function in adherens junction and gap junctions and 14 of these DMGs were differentially expressed. Importantly, among 14 overlapping genes, the *PDGFC* gene was implicated in this study as a potentially important molecule in hair formation, and the validation of the supposition in vitro demonstrates that *PDGFC* plays a significant role in regulating HHIRSC proliferation and migration. The data presented here highlights the importance of epigenetic mechanisms in molding hair shape in Zhongwei fur goats, making their fleece dynamics a promising model for the determination of hair shape and curliness in humans.

## Data Availability Statement

The datasets generated in this paper can be found at Sequence Read Archive: PRJNA524985.

## Ethics Statement

All of the animal experimental procedures were performed in accordance with the guidelines for the care and use of experimental animals established by the Ministry of Agriculture of the People’s Republic of China and approved by the Institutional Animal Care and Use Committee at the Institute of Animal Science, Chinese Academy of Agricultural Sciences.

## Author Contributions

QZ conceived and designed the experiments and revised the manuscript. PX performed the experiments, analyzed the data and wrote the manuscript. TZ and YD analyzed the data and were involved in sample collection. YM revised the manuscript. ZL, WG, XH, YP and LJ participated in the collection of samples. PX and TZ contributed equally.

## Funding

The authors declare that this study received funding from the Science and Technology Innovation Project of the Chinese Academy of Agricultural Sciences (ASTIP-IAS01) and the Modern wool sheep industry system (CARS-39-01). The funders had no role in the study design, data collection and analysis, decision to publish, or preparation of the manuscript.

## Conflict of Interest

The authors declare that the research was conducted in the absence of any commercial or financial relationships that could be construed as a potential conflict of interest.
