# Supplementary material for: Integrated Analysis of Methylome and Transcriptome Changes Reveals the Underlying Regulatory Signatures Driving Curly Wool Transformation in Chinese Zhongwei Goats
Source: Front Genet. 2020 Jan 8;10:1263. doi: 10.3389/fgene.2019.01263 (PMC6960231; doi:10.3389/fgene.2019.01263)
Supplement: Supplementary file 1 [file Image_1.pdf]

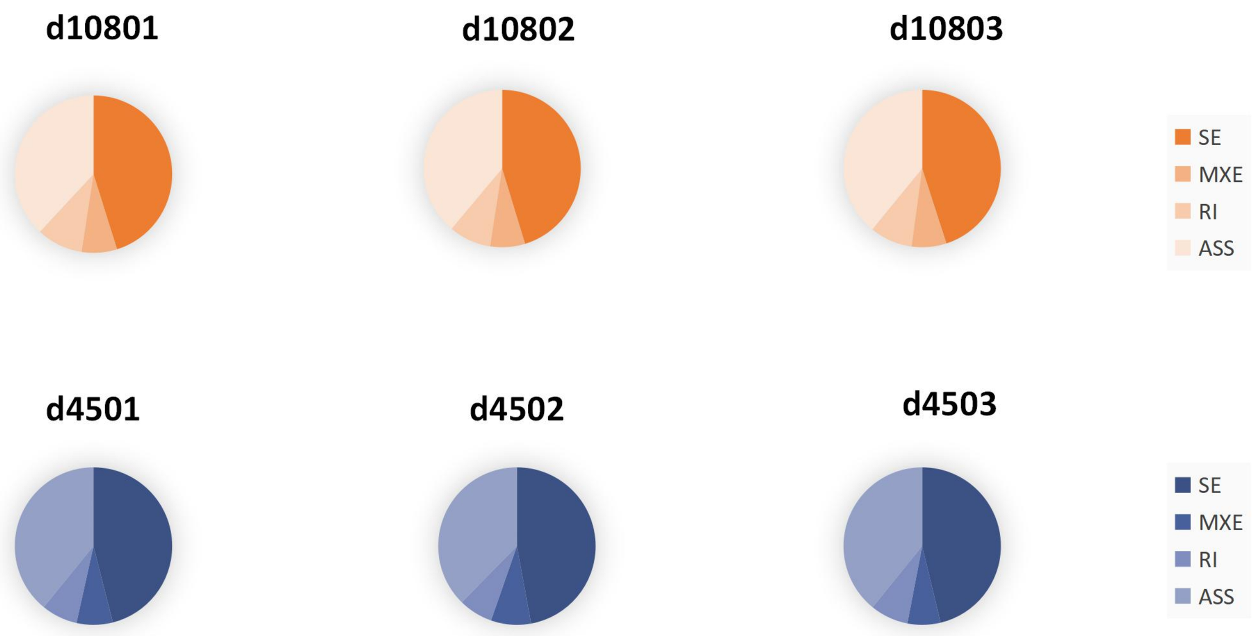

Supplementary Figure 1. Alternative splicing analysis. Pie diagram of forms of alternative splicing events in each sample individual. SE: skipped exon; RI: retained intron; MXE: mutually exclusive exons; ASS: alternative 5' or 3' splice site.
