# Supplementary material for: Integrated Analysis of Methylome and Transcriptome Changes Reveals the Underlying Regulatory Signatures Driving Curly Wool Transformation in Chinese Zhongwei Goats
Source: Front Genet. 2020 Jan 8;10:1263. doi: 10.3389/fgene.2019.01263 (PMC6960231; doi:10.3389/fgene.2019.01263)
Supplement: Supplementary file 2 [file Image_2.pdf]

Blue module

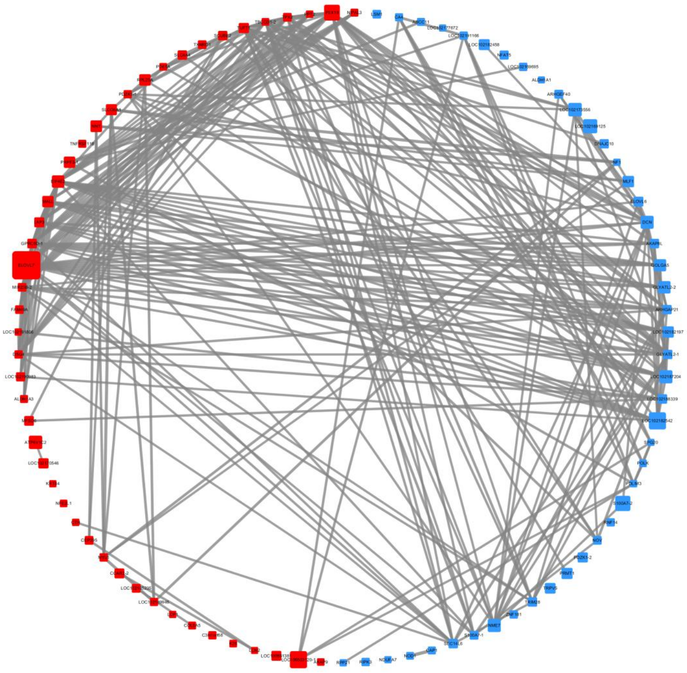

Turquoise module

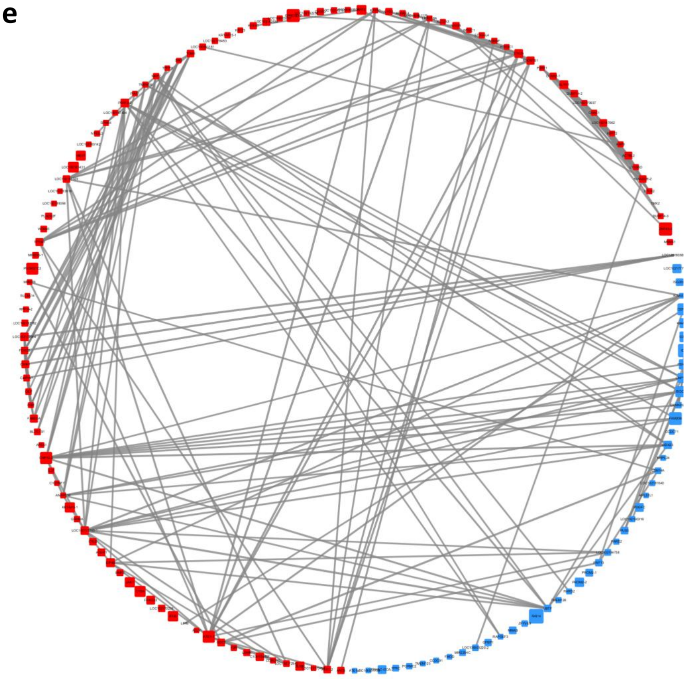

Supplementary Figure 2. WGCNA analysis of DEGs. Squares with blue colors denote downregulated genes in D45, and those with red colors represent upregulated genes in D45. Only correlation edges that weight is greater than 0.4 are retained.
