# Supplementary material for: Integrated Analysis of Methylome and Transcriptome Changes Reveals the Underlying Regulatory Signatures Driving Curly Wool Transformation in Chinese Zhongwei Goats
Source: Front Genet. 2020 Jan 8;10:1263. doi: 10.3389/fgene.2019.01263 (PMC6960231; doi:10.3389/fgene.2019.01263)
Supplement: Supplementary file 3 [file Image_3.pdf]

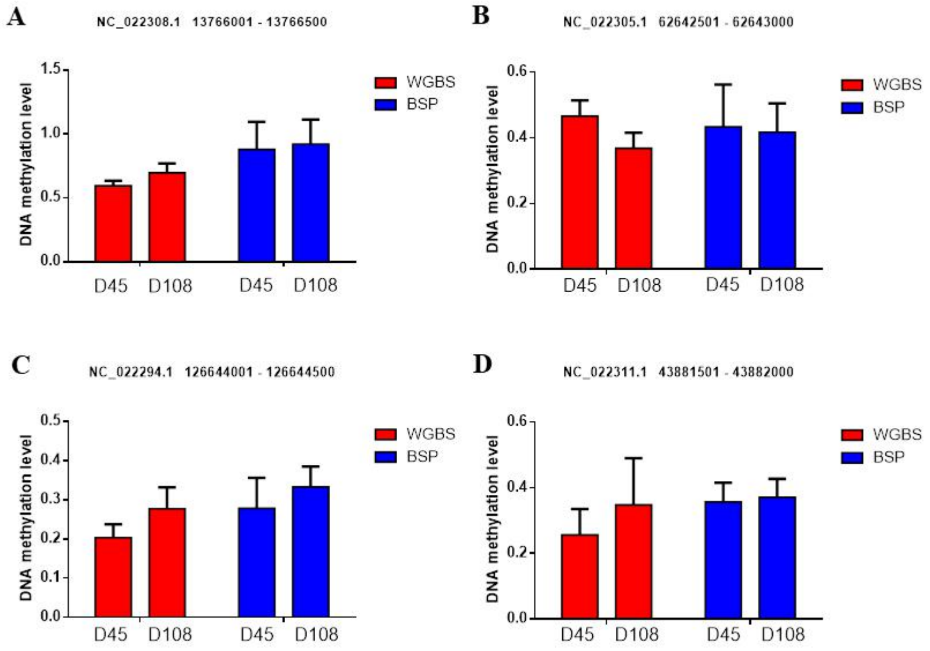

Supplementary Figure 3. DNA methylation level comparison of DMRs between whole genome bisulfite sequencing and bisulfite sequencing PCR.
