# Supplementary material for: Integrated Analysis of Methylome and Transcriptome Changes Reveals the Underlying Regulatory Signatures Driving Curly Wool Transformation in Chinese Zhongwei Goats
Source: Front Genet. 2020 Jan 8;10:1263. doi: 10.3389/fgene.2019.01263 (PMC6960231; doi:10.3389/fgene.2019.01263)
Supplement: Supplementary file 4 [file Image_4.pdf]

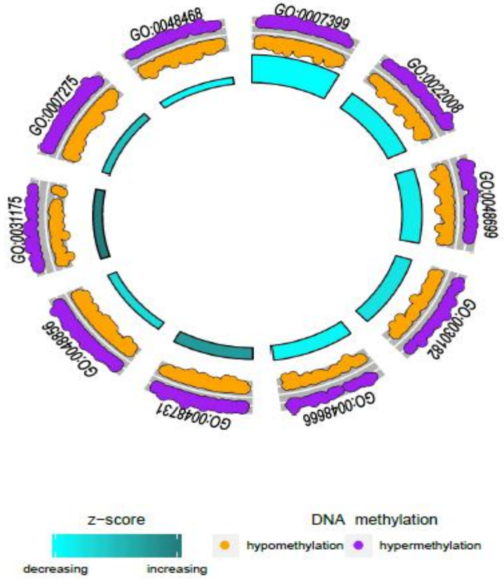

ID	Description
GO:0007399	nervous system development
GO:0022008	neurogenesis
GO:0048699	generation of neurons
GO:0030182	neuron differentiation
GO:0048666	neuron development
GO:0048731	system development
GO:0048856	anatomical structure development
GO:0031175	neuron projection development
GO:0007275	multicellular organism development
GO:0048468	cell development

CC

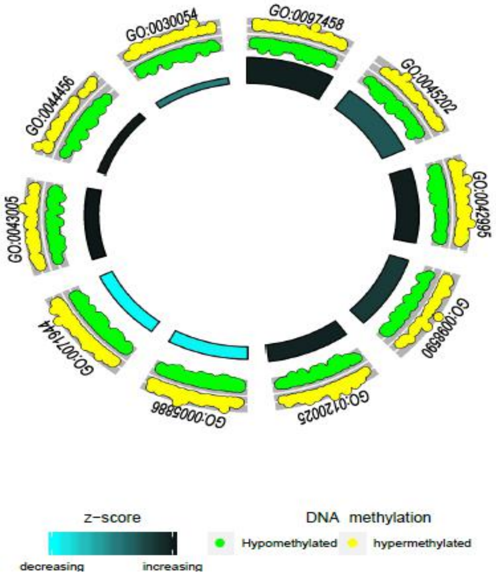

ID	Description
GO:0097458	neuron part
GO:0045202	synapse
GO:0042995	cell projection
GO:0098590	plasma membrane region
GO:0120025	plasma membrane bounded cell projection
GO:0005886	plasma membrane
GO:0071944	cell periphery
GO:0043005	neuron projection
GO:0044456	synapse part
GO:0030054	cell junction

BP

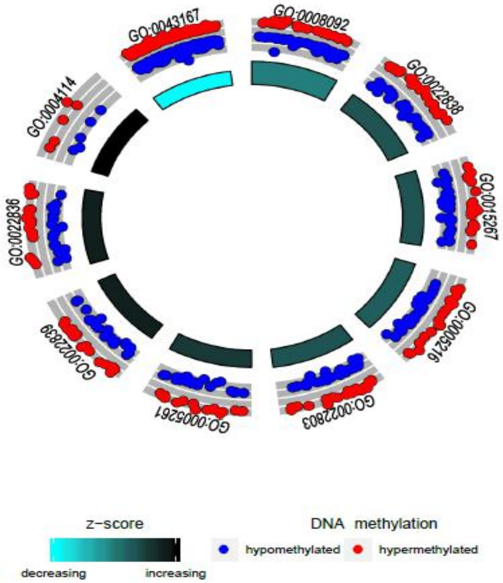

ID	Description
GO:0008092	cytoskeletal protein binding
GO:0022838	substrate−specific channel activity
GO:0015267	channel activity
GO:0005216	ion channel activity
GO:0022803	passive transmembrane transporter activity
GO:0005261	cation channel activity
GO:0022839	ion gated channel activity
GO:0022836	gated channel activity
GO:0004114	3',5'−cyclic−nucleotide phosphodiesterase activity
GO:0043167	ion binding

MF

Supplementary Figure 4. GO terms enrichment analysis of DMGs. The z-score indicates the Difference value between counts of hypermethylated and hypomethylated genes in each GO term.
